# Terabits without fibres

**DOI:** 10.1038/s41377-025-01921-y

**Published:** 2025-07-02

**Authors:** Zhenyu Xie, Qi-Fan Yang

**Affiliations:** 1 State Key Laboratory for Artificial Microstructure and Mesoscopic Physics and Frontiers Science Center for Nano-optoelectronics, School of Physics, Peking University, 100871 Beijing, China; 2 Peking University Yangtze Delta Institute of Optoelectronics, 226010 Nantong, Jiangsu China; 3 Collaborative Innovation Center of Extreme Optics, Shanxi University, 030006 Taiyuan, China

**Keywords:** Applied optics, Frequency combs

## Abstract

A microcomb-based coherent free-space optical link achieves a record-high bandwidth of 8.21 Tbps. Novel beam stabilisation and carrier phase retrieval schemes are employed for turbulence suppression and error correction.

In 1880, Alexander Graham Bell demonstrated speech transmission via a beam of sunlight using his photophone—the forerunner of modern free-space optical (FSO) communication. Contemporary FSO systems employ laser beams to transmit data without physical fibres, supporting rates up to 10 Gbps over distances of approximately 1 km and, in many cases, outperforming conventional radio-based wireless systems^1^. However, challenges including atmospheric turbulence, beam wander, and misalignment continue to restrict link stability and capacity. By contrast, fibre-optic communication remains the backbone of global data infrastructure owing to its low loss and environmental isolation, with recent advances in wavelength-division multiplexing (WDM) and coherent modulation enabling data transmission rates of up to 402 Tbps^2^. Thus, in addition to improving link stability, achieving comparable performance in FSO links requires the adoption of these established fibre-optic techniques.

Microresonator-based optical frequency combs (microcombs) have emerged as a revolutionary technology in this regard^3^. These devices can generate dozens to hundreds of uniformly spaced optical lines from a single continuous-wave laser, with spacings determined by the microresonator’s free spectral range and can be tuned to hundreds of gigahertz to suit WDM applications. Each comb line acts as a coherent carrier, thereby enabling parallel and scalable data transmission. In fibre systems, microcombs have facilitated aggregated bandwidths from the terabit to petabit level using advanced modulation formats^4–7^. Their application in FSO links promises two key advantages: increased channel capacity and enhanced link stability, as the narrow linewidth and high coherence of microcomb carriers can simplify error correction and mitigate the effects of atmospheric absorption and scintillation without adding system complexity^8,9^.

A recent study published in *eLight* by a team led by Professor Chee Wei Wong at the University of California, Los Angeles, demonstrated the feasibility of this approach by achieving an aggregated transmission bandwidth of 8.21 Tbps over an atmospheric link exceeding 160 metres^10^. In this work, a high-power platicon microcomb generated in a Si3N4 microring provided over a hundred high-coherence optical carriers spanning both the C and L bands^11^. Specifically, 28 C-band and 27 L-band carriers were filtered and amplified individually before being modulated using 16QAM IQ modulation in polarisation-diverse mode (PDM) at a symbol rate of 20 GBaud. Following amplification, these channels were transmitted over the free-space link. Despite the challenges posed by atmospheric turbulence and pointing errors inherent to an outdoor configuration, an active beam stabilisation system maintained stable locking with a 10 kHz bandwidth over periods exceeding 10 h. Furthermore, a novel master–slave carrier phase retrieval scheme was employed to simplify the compensation procedures for turbulence-induced intensity fluctuations and pointing errors, thereby reducing the data processing complexity. As a result, the system achieved an aggregated bandwidth of 8.21 Tbps under a PDM-WDM modulation scheme with 55 optical carriers and a total capacity of 5.21 Tbps in single-carrier tests across 35 independent channels.

This demonstration shows that FSO links can attain bandwidths comparable to fibre-optic systems. Moreover, they underscore the promise of microcombs as compact, power-efficient light sources for next-generation optical networks. Free from the constraints of physical fibres, FSO links are well positioned to support a broad range of applications, including optical interconnects^12^, metropolitan access networks^13^, and inter-satellite communications^14^ (Fig. 1).

**Fig. 1:**
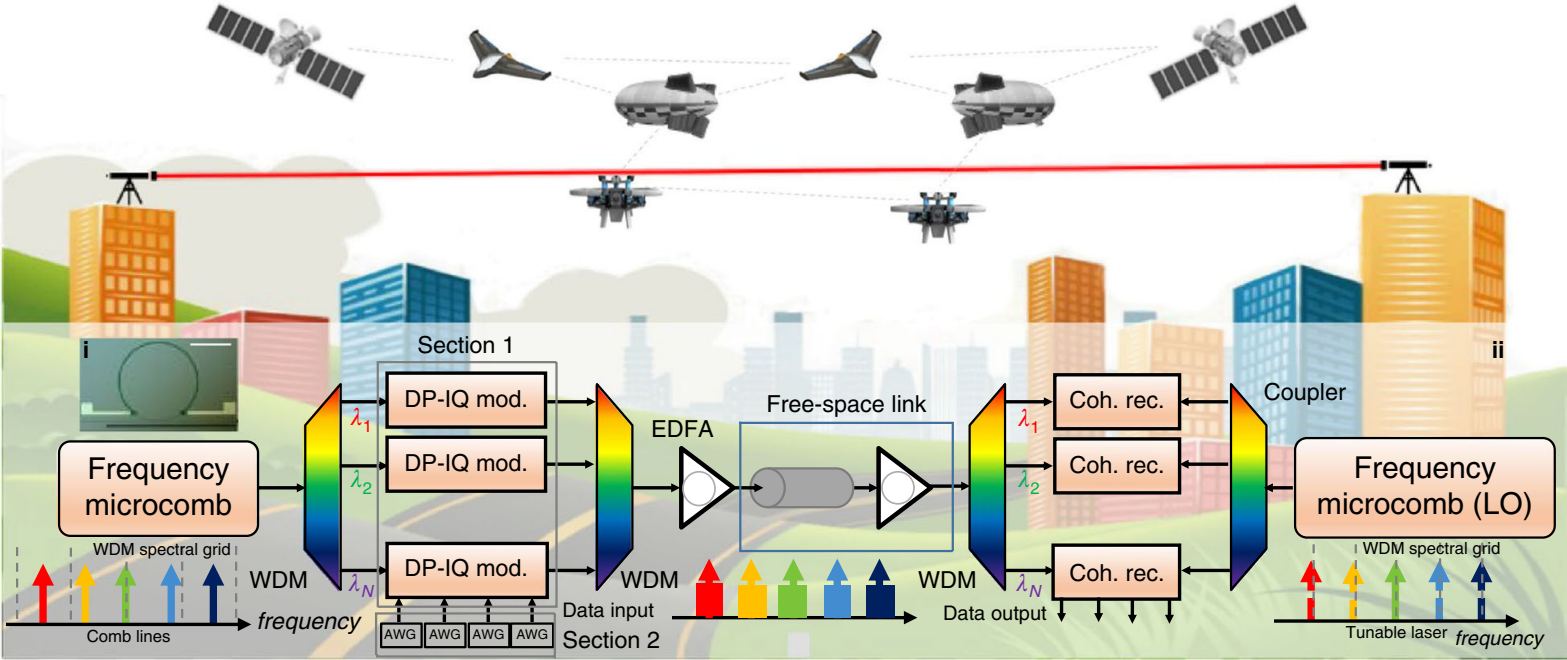
Conceptual illustration of coherent free-space optical communication links based on microcombs. The figure is adapted from ref. ^10^

More than a century after Bell’s pioneering photophone, modern photonics now enables terabit-class data transmission through free space. The integration of microcomb technology and coherent modulation into FSO links shall herald a new era for fibreless communications, offering a promising pathway to meet the growing demands for data bandwidth and extend connectivity beyond physical cabling.
